# Ethyl 4-(2,4-dichloro­phen­yl)-6-(6-meth­oxy-2-naphth­yl)-2-oxocyclo­hex-3-ene-1-carboxyl­ate

**DOI:** 10.1107/S1600536810035130

**Published:** 2010-09-04

**Authors:** William T. A. Harrison, A. N. Mayekar, H. S. Yathirajan, B. Narayana, B. K. Sarojini

**Affiliations:** a Department of Chemistry, University of Aberdeen, Meston Walk, Aberdeen AB24 3UE, Scotland; b Department of Studies in Chemistry, University of Mysore, Manasagangotri, Mysore 570 006, India; c SeQuent Scientific Limited, New Mangalore 575 011, India; d Department of Chemistry, Mangalore University, Mangalagangotri 574 199, India; e Department of Chemistry, P. A. College of Engineering, Nadupadavu, Mangalore 574 153, India

## Abstract

In the title compound, C_26_H_22_Cl_2_O_4_, the cyclo­hexenone ring adopts an approximate half-chair conformation, with two C atoms displaced by −0.485 (6) and 0.218 (6) Å from the plane of the other four ring atoms. The dihedral angles between its four almost coplanar [maximum deviation = 0.006 (2) Å] atoms and the benzene and naphthalene ring systems are 59.26 (13) and 79.94 (9)°, respectively. The dihedral angle between the aromatic rings systems is 77.14 (7)°. A short intra­molecular C—H⋯Cl contact generates an *S*(6) ring. In the crystal, mol­ecules are linked by C—H⋯O and C—H⋯Cl inter­actions to generate a three-dimensional network.

## Related literature

For related structures and background references, see: Li *et al.* (2009*a* ▶,*b* ▶).
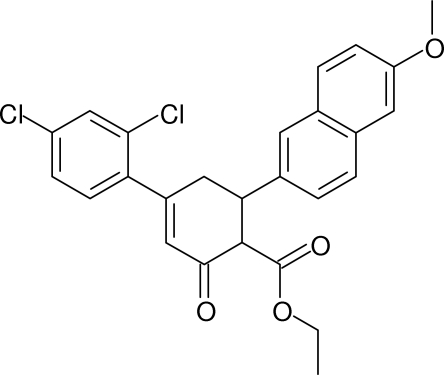


## Experimental

### 

#### Crystal data

C_26_H_22_Cl_2_O_4_
*M* *_r_* = 469.34
Monoclinic, 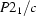

*a* = 14.2156 (4) Å
*b* = 5.8647 (2) Å
*c* = 27.3752 (9) Å
β = 94.840 (2)°
*V* = 2274.14 (13) Å^3^
*Z* = 4
Mo *K*α radiation
μ = 0.32 mm^−1^
*T* = 120 K
0.20 × 0.10 × 0.07 mm

#### Data collection

Nonius KappaCCD diffractometer
24499 measured reflections
5209 independent reflections
3171 reflections with *I* > 2σ(*I*)
*R*_int_ = 0.081

#### Refinement

*R*[*F* ^2^ > 2σ(*F* ^2^)] = 0.062
*wR*(*F* ^2^) = 0.160
*S* = 1.05
5209 reflections
291 parameters
H-atom parameters constrained
Δρ_max_ = 0.39 e Å^−3^
Δρ_min_ = −0.32 e Å^−3^

### 

Data collection: *COLLECT* (Nonius, 1998 ▶); cell refinement: *SCALEPACK* (Otwinowski & Minor, 1997 ▶); data reduction: *DENZO* (Otwinowski & Minor 1997 ▶), *SCALEPACK* and *SORTAV* (Blessing, 1995 ▶); program(s) used to solve structure: *SHELXS97* (Sheldrick, 2008 ▶); program(s) used to refine structure: *SHELXL97* (Sheldrick, 2008 ▶); molecular graphics: *ORTEP-3* (Farrugia, 1997 ▶); software used to prepare material for publication: *SHELXL97*.

## Supplementary Material

Crystal structure: contains datablocks I, global. DOI: 10.1107/S1600536810035130/xu5016sup1.cif

Structure factors: contains datablocks I. DOI: 10.1107/S1600536810035130/xu5016Isup2.hkl

Additional supplementary materials:  crystallographic information; 3D view; checkCIF report

## Figures and Tables

**Table 1:** Hydrogen-bond geometry (Å, °)

*D*—H⋯*A*	*D*—H	H⋯*A*	*D*⋯*A*	*D*—H⋯*A*
C6—H6⋯O1^i^	0.95	2.49	3.411 (4)	164
C8—H8*A*⋯O4^ii^	0.99	2.52	3.388 (4)	146
C8—H8*B*⋯Cl2	0.99	2.69	3.365 (3)	125
C12—H12⋯O1^iii^	0.95	2.42	3.354 (4)	168
C14—H14⋯O4^ii^	0.95	2.33	3.270 (4)	170
C17—H17⋯Cl1^iv^	0.95	2.76	3.635 (3)	153

Symmetry codes: (i) 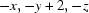; (ii) 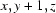; (iii) 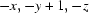; (iv) 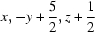.

**Fig. 1.**
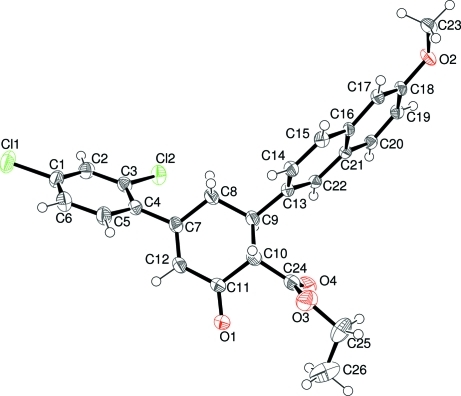
View of the molecular structure of (I) showing 50% displacement ellipsoids (arbitrary spheres for the H atoms).

## supplementary crystallographic information

### Comment

The structure of the title compound, (I), (Fig. 1), was determined as part of our ongoing structural studies (Li *et al.*, 2009*a*,*b*) of substituted cyclohexenones.

The cyclohexenone ring (C7–C12) in (I) adopts an approximate half-chair conformation with C7/C8/C11/C12 statistically coplanar [r.m.s. deviation = 0.0004 Å; individual deviations = 0.0006 (19), -0.0003 (9), 0.0003 (9) and -0.006 (2) Å, respectively] and C9 and C10 displaced from their mean plane by -0.485 (6) and 0.218 (6) Å, respectively. Unlike the equivalent atoms in the related structures ethyl 6-(6-methoxy-2-naphthyl)-4-(4-methylphenyl)-2-oxocyclohex-3- ene-1-carboxylate, (II), (Li *et al.*, 2009*a*) and ethyl 6-(6-methoxy-2-naphthyl)-2-oxo-4-(2-thienyl)cyclohex-3- ene-1-carboxylate, (III), (Li *et al.*, 2009*b*), C9 and C10 in (I) do not display positional disorder. Both atoms are stereogenic centres: in the arbitrarily chosen asymmetric molecule, C9 has *R* configuration and C10 has S, but crystal symmetry generates a racemic mixture of enantiomers.

The dihedral angles between C7/C8/C11/C12 and the benzene (C1–C6) and naphthalene (C13–C22) ring systems are 59.26 (13) and 79.94 (9)°, respectively. The dihedral angle between the aromatic rings systems in (I) is 77.14 (7)°: equivalent values in (II) and (III) are 73.10 (5) and 86.04 (16)°, respectively. The naphthalene ring system (atoms C13–C22) in (I) shows rather high deviations from planarity: the r.m.s. deviation is 0.044Å and maximum deviations are 0.074 (2)Å for C13 and -0.055 (2) for C21. If the two benzene rings (C13/C14/C15/C16/C21/C22 and C16–C21) are considered separately, their r.m.s. deviations are 0.018 and 0.007 Å, respectively, and the dihedral angle between them is 4.85 (16)°. Atom C23 of the terminal methyl group is displaced from the naphthalene ring by 0.466 (4) Å. A short intramolecular C8—H8B···Cl2 contact (Table 1) generates an S(6) ring.

In the crystal, the molecules are linked by C—H···O and C—H···Cl interactions to generate a three-dimensional network.

### Experimental

(2E)-1-(2,4-Dichlorophenyl)-3-(6-methoxy-2-naphthyl)prop-2-en-1-one (1.8 g, 5 mmol) and ethyl acetoacetate (0.65 g, 5 mmol) were refluxed for 4 hr in 15 ml of ethanol in the presence of 0.8 ml 10% NaOH. The mixture was cooled to room temperature and the reaction mass was filtered and recrystallized using acetonitrile to yield colourless blocks of (I) (m.p.: 393–395 K).

### Refinement

The hydrogen atoms were geometrically placed (C—H = 0.95–1.00 Å) and refined as riding with *U*_iso_(H) = 1.2*U*_eq_(C) or 1.5*U*_eq_(methyl C). A rotating rigid-group model was applied to the methyl group.

### Crystal data

**Table:**

C_26_H_22_Cl_2_O_4_	*F*(000) = 976
*M**_r_* = 469.34	*D*_x_ = 1.371 Mg m^−^^3^
Monoclinic, *P*2_1_/*c*	Mo *K*α radiation, λ = 0.71073 Å
Hall symbol: -P 2ybc	Cell parameters from 19625 reflections
*a* = 14.2156 (4) Å	θ = 2.9–27.5°
*b* = 5.8647 (2) Å	µ = 0.32 mm^−^^1^
*c* = 27.3752 (9) Å	*T* = 120 K
β = 94.840 (2)°	Block, colourless
*V* = 2274.14 (13) Å^3^	0.20 × 0.10 × 0.07 mm
*Z* = 4	

### Data collection

**Table:**

Nonius KappaCCD diffractometer	3171 reflections with *I* > 2σ(*I*)
Radiation source: fine-focus sealed tube	*R*_int_ = 0.081
graphite	θ_max_ = 27.5°, θ_min_ = 3.1°
ω and φ scans	*h* = −18→18
24499 measured reflections	*k* = −7→7
5209 independent reflections	*l* = −35→35

### Refinement

**Table:**

Refinement on *F*^2^	Primary atom site location: structure-invariant direct methods
Least-squares matrix: full	Secondary atom site location: difference Fourier map
*R*[*F*^2^ > 2σ(*F*^2^)] = 0.062	Hydrogen site location: inferred from neighbouring sites
*wR*(*F*^2^) = 0.160	H-atom parameters constrained
*S* = 1.05	*w* = 1/[σ^2^(*F*_o_^2^) + (0.0569*P*)^2^ + 1.4128*P*] where *P* = (*F*_o_^2^ + 2*F*_c_^2^)/3
5209 reflections	(Δ/σ)_max_ < 0.001
291 parameters	Δρ_max_ = 0.39 e Å^−^^3^
0 restraints	Δρ_min_ = −0.32 e Å^−^^3^

### Special details

**Table:**

Geometry. All e.s.d.'s (except the e.s.d. in the dihedral angle between two l.s. planes) are estimated using the full covariance matrix. The cell e.s.d.'s are taken into account individually in the estimation of e.s.d.'s in distances, angles and torsion angles; correlations between e.s.d.'s in cell parameters are only used when they are defined by crystal symmetry. An approximate (isotropic) treatment of cell e.s.d.'s is used for estimating e.s.d.'s involving l.s. planes.
Refinement. Refinement of *F*^2^ against ALL reflections. The weighted *R*-factor *wR* and goodness of fit *S* are based on *F*^2^, conventional *R*-factors *R* are based on *F*, with *F* set to zero for negative *F*^2^. The threshold expression of *F*^2^ > σ(*F*^2^) is used only for calculating *R*-factors(gt) *etc*. and is not relevant to the choice of reflections for refinement. *R*-factors based on *F*^2^ are statistically about twice as large as those based on *F*, and *R*- factors based on ALL data will be even larger.

### Fractional atomic coordinates and isotropic or equivalent isotropic displacement parameters (Å^2^)

**Table:**

	*x*	*y*	*z*	*U*_iso_*/*U*_eq_	
C1	0.2743 (2)	1.2107 (5)	−0.05812 (11)	0.0325 (7)	
C2	0.3364 (2)	1.0439 (5)	−0.04033 (11)	0.0317 (7)	
H2	0.3962	1.0277	−0.0530	0.038*	
C3	0.3101 (2)	0.9003 (5)	−0.00372 (10)	0.0271 (7)	
C4	0.2229 (2)	0.9190 (5)	0.01610 (10)	0.0268 (7)	
C5	0.1630 (2)	1.0922 (6)	−0.00319 (11)	0.0337 (8)	
H5	0.1031	1.1100	0.0093	0.040*	
C6	0.1876 (2)	1.2388 (6)	−0.03978 (12)	0.0364 (8)	
H6	0.1457	1.3558	−0.0519	0.044*	
C7	0.18917 (19)	0.7672 (5)	0.05408 (10)	0.0256 (7)	
C8	0.2417 (2)	0.7606 (5)	0.10417 (10)	0.0279 (7)	
H8A	0.2294	0.9038	0.1217	0.033*	
H8B	0.3103	0.7529	0.1005	0.033*	
C9	0.21375 (19)	0.5572 (5)	0.13528 (10)	0.0272 (7)	
H9	0.2380	0.4157	0.1203	0.033*	
C10	0.10628 (19)	0.5373 (6)	0.13368 (11)	0.0304 (7)	
H10	0.0810	0.6780	0.1486	0.036*	
C11	0.0638 (2)	0.5189 (6)	0.08069 (11)	0.0312 (7)	
C12	0.1079 (2)	0.6548 (5)	0.04425 (11)	0.0311 (7)	
H12	0.0774	0.6639	0.0121	0.037*	
C13	0.26075 (19)	0.5753 (5)	0.18738 (10)	0.0256 (7)	
C14	0.24668 (19)	0.7727 (5)	0.21574 (10)	0.0274 (7)	
H14	0.2040	0.8869	0.2031	0.033*	
C15	0.29374 (19)	0.8015 (5)	0.26125 (10)	0.0249 (6)	
H15	0.2819	0.9335	0.2799	0.030*	
C16	0.35953 (18)	0.6376 (5)	0.28076 (9)	0.0203 (6)	
C17	0.41590 (18)	0.6728 (5)	0.32532 (10)	0.0220 (6)	
H17	0.4088	0.8083	0.3437	0.026*	
C18	0.48025 (19)	0.5124 (5)	0.34184 (10)	0.0232 (6)	
C19	0.49063 (19)	0.3078 (5)	0.31567 (10)	0.0239 (6)	
H19	0.5351	0.1965	0.3280	0.029*	
C20	0.43709 (19)	0.2697 (5)	0.27280 (10)	0.0237 (6)	
H20	0.4443	0.1311	0.2556	0.028*	
C21	0.37076 (18)	0.4340 (5)	0.25358 (10)	0.0213 (6)	
C22	0.31971 (19)	0.4083 (5)	0.20690 (10)	0.0235 (6)	
H22	0.3267	0.2719	0.1889	0.028*	
C23	0.5480 (2)	0.7485 (5)	0.40688 (11)	0.0318 (7)	
H23A	0.5984	0.7451	0.4336	0.048*	
H23B	0.4879	0.7852	0.4202	0.048*	
H23C	0.5624	0.8647	0.3829	0.048*	
C24	0.0723 (2)	0.3334 (6)	0.16028 (11)	0.0324 (7)	
C25	−0.0480 (3)	0.1826 (8)	0.20559 (15)	0.0657 (12)	
H25A	−0.0850	0.2342	0.2326	0.079*	
H25B	0.0014	0.0764	0.2195	0.079*	
C26	−0.1126 (3)	0.0587 (8)	0.16739 (18)	0.0768 (14)	
H26A	−0.1457	−0.0640	0.1832	0.115*	
H26B	−0.0750	−0.0062	0.1424	0.115*	
H26C	−0.1588	0.1662	0.1519	0.115*	
O1	−0.00458 (15)	0.3976 (4)	0.06997 (8)	0.0436 (6)	
O2	0.54118 (13)	0.5310 (3)	0.38362 (7)	0.0301 (5)	
O3	−0.00269 (15)	0.3819 (4)	0.18412 (9)	0.0468 (6)	
O4	0.10805 (16)	0.1490 (4)	0.15843 (9)	0.0468 (6)	
Cl1	0.30566 (6)	1.39074 (16)	−0.10447 (3)	0.0484 (3)	
Cl2	0.38884 (5)	0.68373 (14)	0.01551 (3)	0.0360 (2)	

### Atomic displacement parameters (Å^2^)

**Table:**

	*U*^11^	*U*^22^	*U*^33^	*U*^12^	*U*^13^	*U*^23^
C1	0.0409 (18)	0.0292 (18)	0.0274 (17)	−0.0051 (15)	0.0038 (14)	0.0047 (14)
C2	0.0321 (16)	0.0368 (19)	0.0272 (16)	−0.0022 (15)	0.0085 (13)	−0.0014 (15)
C3	0.0288 (15)	0.0312 (17)	0.0210 (15)	0.0002 (13)	0.0014 (12)	−0.0018 (13)
C4	0.0297 (15)	0.0310 (17)	0.0193 (15)	−0.0038 (14)	0.0005 (12)	0.0004 (13)
C5	0.0286 (16)	0.043 (2)	0.0297 (17)	0.0020 (15)	0.0037 (13)	0.0088 (15)
C6	0.0388 (18)	0.0384 (19)	0.0318 (18)	0.0057 (15)	0.0021 (15)	0.0072 (15)
C7	0.0262 (15)	0.0312 (17)	0.0195 (15)	0.0029 (13)	0.0025 (12)	0.0003 (13)
C8	0.0254 (15)	0.0380 (18)	0.0198 (15)	0.0001 (14)	−0.0003 (12)	0.0000 (13)
C9	0.0273 (15)	0.0326 (18)	0.0211 (15)	−0.0001 (13)	−0.0006 (12)	0.0021 (13)
C10	0.0255 (15)	0.0407 (19)	0.0247 (16)	−0.0023 (14)	0.0008 (13)	0.0023 (14)
C11	0.0257 (15)	0.0404 (19)	0.0265 (17)	−0.0059 (15)	−0.0041 (13)	0.0032 (15)
C12	0.0301 (16)	0.0409 (19)	0.0215 (15)	−0.0027 (15)	−0.0026 (13)	0.0041 (14)
C13	0.0266 (15)	0.0308 (17)	0.0190 (15)	−0.0062 (13)	0.0002 (12)	0.0028 (13)
C14	0.0251 (15)	0.0311 (17)	0.0254 (16)	0.0000 (13)	−0.0009 (13)	0.0041 (14)
C15	0.0249 (14)	0.0259 (16)	0.0241 (15)	0.0023 (13)	0.0036 (12)	−0.0004 (13)
C16	0.0198 (13)	0.0254 (16)	0.0157 (13)	−0.0001 (12)	0.0020 (11)	0.0037 (12)
C17	0.0241 (14)	0.0233 (15)	0.0186 (14)	0.0013 (12)	0.0015 (11)	−0.0012 (12)
C18	0.0245 (14)	0.0294 (16)	0.0156 (14)	−0.0020 (13)	0.0015 (11)	0.0011 (13)
C19	0.0260 (14)	0.0231 (16)	0.0226 (15)	0.0009 (13)	0.0017 (12)	0.0010 (13)
C20	0.0285 (15)	0.0214 (16)	0.0218 (15)	−0.0022 (12)	0.0051 (12)	−0.0011 (12)
C21	0.0210 (14)	0.0238 (16)	0.0194 (14)	−0.0037 (12)	0.0033 (11)	0.0010 (12)
C22	0.0262 (14)	0.0252 (16)	0.0194 (14)	−0.0062 (13)	0.0027 (12)	−0.0005 (13)
C23	0.0350 (17)	0.0341 (18)	0.0250 (16)	−0.0030 (14)	−0.0046 (13)	−0.0031 (14)
C24	0.0263 (16)	0.041 (2)	0.0288 (17)	−0.0013 (15)	−0.0035 (13)	0.0014 (16)
C25	0.062 (3)	0.081 (3)	0.058 (3)	−0.017 (2)	0.029 (2)	0.015 (2)
C26	0.055 (3)	0.074 (3)	0.104 (4)	−0.025 (2)	0.019 (3)	−0.006 (3)
O1	0.0360 (12)	0.0607 (16)	0.0325 (13)	−0.0196 (12)	−0.0070 (10)	0.0123 (12)
O2	0.0363 (12)	0.0296 (12)	0.0221 (11)	0.0050 (9)	−0.0103 (9)	−0.0021 (9)
O3	0.0386 (13)	0.0583 (16)	0.0455 (14)	−0.0024 (12)	0.0160 (11)	−0.0035 (13)
O4	0.0385 (13)	0.0447 (16)	0.0560 (16)	0.0066 (12)	−0.0034 (12)	0.0070 (13)
Cl1	0.0578 (6)	0.0480 (6)	0.0407 (5)	−0.0047 (4)	0.0109 (4)	0.0188 (4)
Cl2	0.0380 (4)	0.0395 (5)	0.0312 (4)	0.0087 (4)	0.0071 (3)	0.0055 (4)

### Geometric parameters (Å, °)

**Table:**

C1—C2	1.378 (4)	C14—H14	0.9500
C1—C6	1.380 (4)	C15—C16	1.414 (4)
C1—Cl1	1.737 (3)	C15—H15	0.9500
C2—C3	1.384 (4)	C16—C17	1.417 (4)
C2—H2	0.9500	C16—C21	1.423 (4)
C3—C4	1.399 (4)	C17—C18	1.362 (4)
C3—Cl2	1.745 (3)	C17—H17	0.9500
C4—C5	1.400 (4)	C18—O2	1.379 (3)
C4—C7	1.479 (4)	C18—C19	1.412 (4)
C5—C6	1.387 (4)	C19—C20	1.362 (4)
C5—H5	0.9500	C19—H19	0.9500
C6—H6	0.9500	C20—C21	1.418 (4)
C7—C12	1.338 (4)	C20—H20	0.9500
C7—C8	1.506 (4)	C21—C22	1.423 (4)
C8—C9	1.537 (4)	C22—H22	0.9500
C8—H8A	0.9900	C23—O2	1.425 (3)
C8—H8B	0.9900	C23—H23A	0.9800
C9—C13	1.527 (4)	C23—H23B	0.9800
C9—C10	1.529 (4)	C23—H23C	0.9800
C9—H9	1.0000	C24—O4	1.198 (4)
C10—C24	1.501 (4)	C24—O3	1.328 (4)
C10—C11	1.528 (4)	C25—O3	1.480 (4)
C10—H10	1.0000	C25—C26	1.517 (6)
C11—O1	1.220 (3)	C25—H25A	0.9900
C11—C12	1.459 (4)	C25—H25B	0.9900
C12—H12	0.9500	C26—H26A	0.9800
C13—C22	1.368 (4)	C26—H26B	0.9800
C13—C14	1.417 (4)	C26—H26C	0.9800
C14—C15	1.374 (4)		
			
C2—C1—C6	121.4 (3)	C15—C14—H14	119.4
C2—C1—Cl1	119.6 (2)	C13—C14—H14	119.4
C6—C1—Cl1	119.0 (2)	C14—C15—C16	121.0 (3)
C1—C2—C3	118.9 (3)	C14—C15—H15	119.5
C1—C2—H2	120.5	C16—C15—H15	119.5
C3—C2—H2	120.5	C15—C16—C17	122.3 (3)
C2—C3—C4	122.4 (3)	C15—C16—C21	118.2 (2)
C2—C3—Cl2	117.1 (2)	C17—C16—C21	119.5 (2)
C4—C3—Cl2	120.5 (2)	C18—C17—C16	120.0 (3)
C3—C4—C5	116.2 (3)	C18—C17—H17	120.0
C3—C4—C7	125.2 (3)	C16—C17—H17	120.0
C5—C4—C7	118.6 (3)	C17—C18—O2	125.3 (3)
C6—C5—C4	122.7 (3)	C17—C18—C19	120.9 (2)
C6—C5—H5	118.7	O2—C18—C19	113.8 (2)
C4—C5—H5	118.7	C20—C19—C18	120.1 (3)
C1—C6—C5	118.4 (3)	C20—C19—H19	119.9
C1—C6—H6	120.8	C18—C19—H19	119.9
C5—C6—H6	120.8	C19—C20—C21	120.9 (3)
C12—C7—C4	118.7 (3)	C19—C20—H20	119.5
C12—C7—C8	121.6 (3)	C21—C20—H20	119.5
C4—C7—C8	119.3 (2)	C20—C21—C16	118.5 (2)
C7—C8—C9	113.1 (2)	C20—C21—C22	122.3 (3)
C7—C8—H8A	109.0	C16—C21—C22	119.2 (2)
C9—C8—H8A	109.0	C13—C22—C21	121.6 (3)
C7—C8—H8B	109.0	C13—C22—H22	119.2
C9—C8—H8B	109.0	C21—C22—H22	119.2
H8A—C8—H8B	107.8	O2—C23—H23A	109.5
C13—C9—C10	112.9 (2)	O2—C23—H23B	109.5
C13—C9—C8	110.5 (2)	H23A—C23—H23B	109.5
C10—C9—C8	110.4 (2)	O2—C23—H23C	109.5
C13—C9—H9	107.6	H23A—C23—H23C	109.5
C10—C9—H9	107.6	H23B—C23—H23C	109.5
C8—C9—H9	107.6	O4—C24—O3	125.1 (3)
C24—C10—C11	106.7 (2)	O4—C24—C10	123.0 (3)
C24—C10—C9	114.1 (2)	O3—C24—C10	111.9 (3)
C11—C10—C9	110.3 (2)	O3—C25—C26	111.3 (3)
C24—C10—H10	108.6	O3—C25—H25A	109.4
C11—C10—H10	108.6	C26—C25—H25A	109.4
C9—C10—H10	108.6	O3—C25—H25B	109.4
O1—C11—C12	122.2 (3)	C26—C25—H25B	109.4
O1—C11—C10	120.8 (3)	H25A—C25—H25B	108.0
C12—C11—C10	116.9 (2)	C25—C26—H26A	109.5
C7—C12—C11	123.0 (3)	C25—C26—H26B	109.5
C7—C12—H12	118.5	H26A—C26—H26B	109.5
C11—C12—H12	118.5	C25—C26—H26C	109.5
C22—C13—C14	118.7 (3)	H26A—C26—H26C	109.5
C22—C13—C9	121.4 (3)	H26B—C26—H26C	109.5
C14—C13—C9	119.8 (3)	C18—O2—C23	117.1 (2)
C15—C14—C13	121.1 (3)	C24—O3—C25	114.9 (3)
			
C6—C1—C2—C3	0.8 (5)	C8—C9—C13—C22	−120.9 (3)
Cl1—C1—C2—C3	−179.4 (2)	C10—C9—C13—C14	−67.7 (3)
C1—C2—C3—C4	−0.3 (4)	C8—C9—C13—C14	56.4 (3)
C1—C2—C3—Cl2	177.1 (2)	C22—C13—C14—C15	2.5 (4)
C2—C3—C4—C5	0.0 (4)	C9—C13—C14—C15	−174.9 (3)
Cl2—C3—C4—C5	−177.3 (2)	C13—C14—C15—C16	1.6 (4)
C2—C3—C4—C7	178.0 (3)	C14—C15—C16—C17	173.6 (3)
Cl2—C3—C4—C7	0.7 (4)	C14—C15—C16—C21	−4.4 (4)
C3—C4—C5—C6	−0.1 (5)	C15—C16—C17—C18	−178.1 (3)
C7—C4—C5—C6	−178.3 (3)	C21—C16—C17—C18	0.0 (4)
C2—C1—C6—C5	−1.0 (5)	C16—C17—C18—O2	177.6 (2)
Cl1—C1—C6—C5	179.2 (2)	C16—C17—C18—C19	−1.3 (4)
C4—C5—C6—C1	0.6 (5)	C17—C18—C19—C20	1.1 (4)
C3—C4—C7—C12	−122.5 (3)	O2—C18—C19—C20	−177.9 (2)
C5—C4—C7—C12	55.4 (4)	C18—C19—C20—C21	0.4 (4)
C3—C4—C7—C8	63.4 (4)	C19—C20—C21—C16	−1.7 (4)
C5—C4—C7—C8	−118.6 (3)	C19—C20—C21—C22	174.4 (3)
C12—C7—C8—C9	20.0 (4)	C15—C16—C21—C20	179.6 (2)
C4—C7—C8—C9	−166.2 (3)	C17—C16—C21—C20	1.4 (4)
C7—C8—C9—C13	−173.5 (2)	C15—C16—C21—C22	3.4 (4)
C7—C8—C9—C10	−47.9 (3)	C17—C16—C21—C22	−174.8 (2)
C13—C9—C10—C24	−59.8 (3)	C14—C13—C22—C21	−3.5 (4)
C8—C9—C10—C24	176.0 (3)	C9—C13—C22—C21	173.9 (2)
C13—C9—C10—C11	−179.8 (3)	C20—C21—C22—C13	−175.5 (3)
C8—C9—C10—C11	56.0 (3)	C16—C21—C22—C13	0.6 (4)
C24—C10—C11—O1	19.1 (4)	C11—C10—C24—O4	81.0 (4)
C9—C10—C11—O1	143.5 (3)	C9—C10—C24—O4	−41.0 (4)
C24—C10—C11—C12	−161.8 (3)	C11—C10—C24—O3	−96.8 (3)
C9—C10—C11—C12	−37.4 (4)	C9—C10—C24—O3	141.2 (3)
C4—C7—C12—C11	−173.8 (3)	C17—C18—O2—C23	−10.7 (4)
C8—C7—C12—C11	0.1 (5)	C19—C18—O2—C23	168.3 (2)
O1—C11—C12—C7	−171.8 (3)	O4—C24—O3—C25	−5.8 (5)
C10—C11—C12—C7	9.1 (5)	C10—C24—O3—C25	172.0 (3)
C10—C9—C13—C22	114.9 (3)	C26—C25—O3—C24	−81.2 (4)

### Hydrogen-bond geometry (Å, °)

**Table:**

*D*—H···*A*	*D*—H	H···*A*	*D*···*A*	*D*—H···*A*
C6—H6···O1^i^	0.95	2.49	3.411 (4)	164
C8—H8A···O4^ii^	0.99	2.52	3.388 (4)	146
C8—H8B···Cl2	0.99	2.69	3.365 (3)	125
C12—H12···O1^iii^	0.95	2.42	3.354 (4)	168
C14—H14···O4^ii^	0.95	2.33	3.270 (4)	170
C17—H17···Cl1^iv^	0.95	2.76	3.635 (3)	153

Symmetry codes: (i) −*x*, −*y*+2, −*z*; (ii) *x*, *y*+1, *z*; (iii) −*x*, −*y*+1, −*z*; (iv) *x*, −*y*+5/2, *z*+1/2.
